# Bilateral hand-restricted drug eruption induced by blonanserin: a case report

**DOI:** 10.3389/fpsyt.2026.1751750

**Published:** 2026-03-05

**Authors:** Liang Lv, Meng-Yun Guan, Xiao-Yu Zhang

**Affiliations:** Department of Psychiatry, HuzhouThird Municipal Hospital, Huzhou, Zhejiang, China

**Keywords:** adverse drug reaction, antipsychotic agents, blonanserin, case report, drug eruptions, hand

## Abstract

**Background:**

Cutaneous adverse drug reactions due to antipsychotic therapy are well-documented, although their manifestations are highly variable. Blonanserin, a second-generation antipsychotic, is generally considered to have a favorable tolerability profile. Cutaneous reactions to blonanserin are rare and, more importantly, remain poorly characterized. This case report describes a novel and distinctive presentation of a bilateral hand-restricted drug eruption attributed to blonanserin.

**Case Summary:**

A 26-year-old woman without prior psychiatric history was hospitalized for an acute psychotic episode. Treatment was initiated with quetiapine (25 mg at bedtime), and blonanserin (8 mg/day) was added on the fifth hospital day to accelerate symptom control. On the fourth day of combination therapy, the patient developed symmetrical, erythematous papules limited to the dorsal and palmar aspects of both hands, with no history of new topical exposures. Although the rash was initially managed as dyshidrotic eczema or contact dermatitis with antihistamines and potent topical corticosteroids, it worsened over the following week. Given the strong temporal relationship, blonanserin was discontinued on the tenth day after rash onset, while quetiapine was continued. A dramatic improvement was observed within 72 hours, with near-complete resolution within one week. The patient’s psychiatric symptoms remained stable on quetiapine monotherapy.

**Conclusion:**

This is the first report of a bilateral hand-limited drug eruption due to blonanserin and underscores that prompt withdrawal upon rash appearance is important for patient comfort and aligns with good clinical practice.

## 1 Introduction

Antipsychotic drugs, including typical and atypical agents, are associated with various cutaneous adverse drug reactions (CADRs) (1, 2). Although their overall prevalence has been estimated to be around 5% (3), the severity of these reactions can range widely from mild manifestations to major life-threatening events (4). Among them, blonanserin, a novel atypical antipsychotic, exerts its efficacy through high selective antagonism of dopamine D_2_ and serotonin 5-HT_2_A receptors. It is well-tolerated with manageable metabolic side effects, and can be widely used in female patients aged 18–40 years with schizophrenia (5, 6). The most common adverse reactions of blonanserin are akathisis, insomnia, and elevated blood prolactin levels (7). However, the specific phenotypic spectrum and detailed clinical characteristics of blonanserin-associated CADRs remain poorly characterized in the literature, with only sparse reports available that lack comprehensive description. We hereby report a distinctive case of a bilateral hand-restricted drug eruption attributed to blonanserin, aiming to better delineate this potential adverse event.

## 2 Case presentation

### 2.1 Chief complaints

A 26-year-old female was admitted to the hospital due to 4 days of sleep disturbance, incoherent speech, and auditory hallucinations.

### 2.2 History of present illness

**Figure 1:**
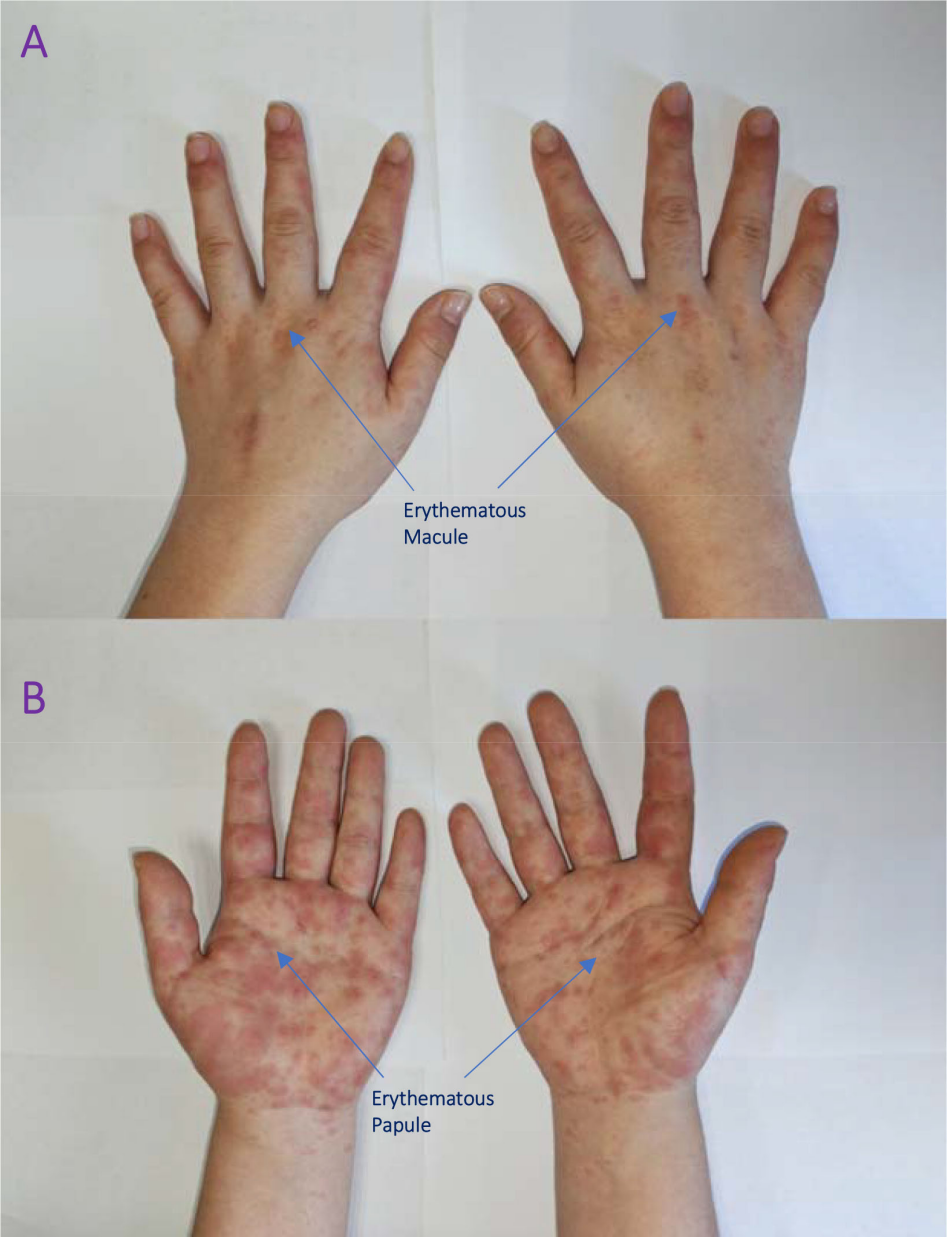
Bilateral hand rash on the day of onset. **(A)** Dorsal view of both hands with symmetrical, erythematous papules in a patchy distribution; **(B)** Palmar view with similar involvement.

The patient presented with acute-onset psychosis, potentially triggered by emotional stress. Symptoms included insomnia, talking to herself, auditory hallucinations (hearing voices), delusions, emotional instability, and wandering behavior. There was no previous psychiatric history or treatment. The initial admission diagnosis was acute schizophrenia-like psychotic disorder. Treatment with quetiapine at 25 mg/day was initiated, which was gradually titrated up to 100 mg/day over 8 days. To augment antipsychotic treatment, blonanserin (8 mg/day) was added on the fifth hospital day. On the fourth day of combination therapy (hospital day 9), the patient developed symmetric, erythematous, papular eruptions on the dorsal and palmar aspects of both hands. The lesions first appeared as ill-defined, bright red macules which subsequently coalesced and evolved into well-demarcated, red papules. The patient did not report pruritus. From the outset, the eruption was strictly confined to the hands in a symmetrical distribution. Over the following week, despite symptomatic management (detailed below), the rash progressed: the erythema darkened to a deep red color, existing papules increased in number and became confluent, and fine scaling developed on the fingertips. Importantly, the rash did not extend beyond the wrists to the forearms or any other body site. The patient denied recent use of new hand creams, detergents, or topical medications, and had no occupational exposure history. The rash was documented photographically (**Figure 1**), showing erythematous papules on both hands, with a patchy distribution.

### 2.3 History of past illness

The patient denied other illnesses. She reported a penicillin allergy.

### 2.4 Personal and family history

The patient did not have a history of substance abuse. Her mother had a family history of depression.

### 2.5 Physical examination

Vital signs were stable on admission. General physical and neurological examinations were unremarkable.

### 2.6 Systemic assessment following rash onset

After the appearance of the cutaneous eruption, the patient was specifically evaluated for signs and symptoms indicative of a systemic drug reaction or severe cutaneous adverse reaction (SCAR). She remained afebrile (temperature <37.3 °C), and no lymphadenopathy was detected in the cervical, axillary, or inguinal regions. She denied odynophagia (painful swallowing), malaise, fatigue, or involvement of the oral, ocular, or genital mucosa. Her vital signs remained stable throughout this period.

### 2.7 Laboratory examinations

Routine laboratory tests performed at admission and during the rash were unremarkable, showing no eosinophilia. As the clinical presentation was highly suggestive of a localized drug eruption temporally linked to blonanserin, further allergological or inflammatory marker testing was not pursued.

### 2.8 Imaging examinations

Chest X-ray performed during the initial workup for acute psychosis showed no abnormalities.

### 2.9 Multidisciplinary expert consultation

**Figure 2:**
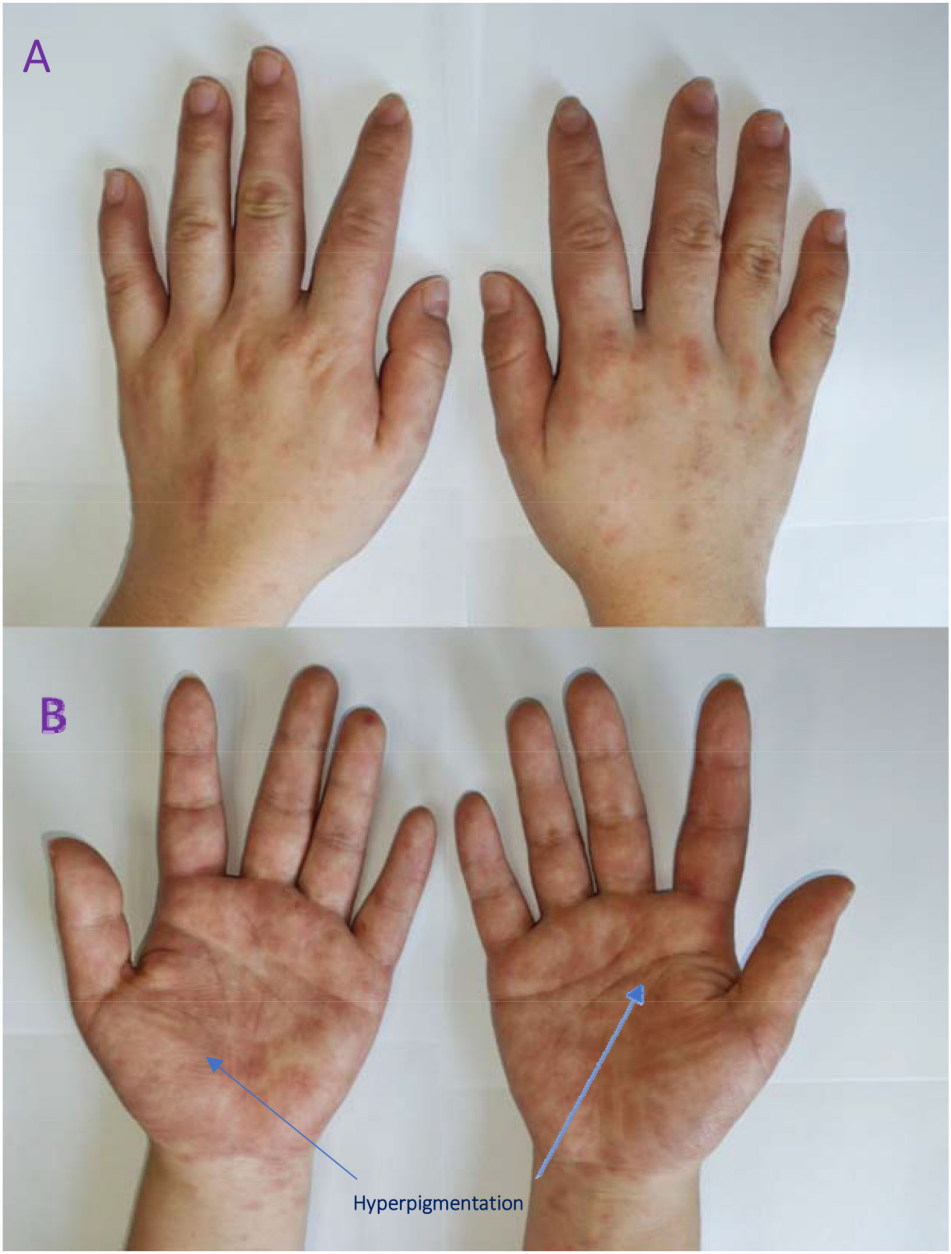
Progression of the rash on day 10. Darkening in color and expansion in areas of the lesions compared to **Figure 1**.

Dermatology consultations were obtained on day 1 and day 6 after rash onset. The consulting dermatologists considered primary differential diagnoses of dyshidrotic eczema and allergic contact dermatitis. Accordingly, a trial of symptomatic therapy was initiated with oral loratadine 10 mg once daily and topical triamcinolone acetonide and econazole cream applied twice daily to the affected areas. Despite this targeted regimen over the subsequent 5 days, the rash not only showed no improvement but progressively worsened (**Figure 2**). The lack of response to potent topical steroids, which would typically ameliorate eczematous or contact dermatitis, became a key point against these initial diagnoses.

## 3 Diagnosis

The persistence of the eruption strictly confined to the bilateral hands, combined with its lack of response to potent topical corticosteroid therapy, was pivotal in arriving at the diagnosis of a drug-induced eruption. While the hands are a common site for contact dermatitis, the symmetrical onset and the absence of a clear exposure history made this less likely. More importantly, the failure to improve with potent topical steroid therapy (triamcinolone acetonide) argued strongly against primary inflammatory dermatoses such as dyshidrotic eczema or allergic contact dermatitis, which typically show at least partial response to such treatment. Having thus discounted the most common localized causes, and given the strong temporal association with the recent introduction of blonanserin (with no other new systemic medications, foods, or personal care products), a localized, drug-induced eruption emerged as the most probable explanation. This diagnostic logic supported the decision to discontinue blonanserin as a targeted diagnostic and therapeutic intervention.

Combined with the patient’s medical history and the observed therapeutic course, the final diagnoses were an acute schizophrenia-like psychotic disorder and a drug-induced rash localized to the hands, secondary to blonanserin.

## 4 Treatment

Blonanserin was discontinued on the 10th day following onset of the hand rash. Quetiapine was continued as the antipsychotic therapy, and the dose was increased to 150 mg at night. The detailed timeline is summarized in **Table 1**.

**Table 1:** Timeline of drug therapy and skin reactions relative to rash onset.

Time relative to rash onset	Medication regimen	Skin changes and management
Day -4	Blonanserin (BLON) introduced at 4 mg twice daily (8 mg/day). Quetiapine (QUE) continued.	Baseline, no rash.
Day 0 (rash onset)	QUE 100 mg daily + BLON 4 mg twice daily.	Symmetrical, erythematous papules appeared on the dorsal and palmar aspects of both hands.
Day 1	QUE 100 mg daily + BLON 4 mg twice daily.	First dermatology consultation. Oral ebastine (EBA) 10 mg daily started.
Day 2	QUE 100 mg daily + BLON 4 mg twice daily.	Rash darkened in color and expanded in area.
Day 6	QUE 100 mg daily + BLON 4 mg twice daily.	Second dermatology consultation. Topical triamcinolone acetonide and econazole (TAE) cream twice daily added.
Day 10 (key decision)	BLON discontinued. QUE dose increased to 150 mg daily.	Rash persisted and worsened despite treatment. Decision to discontinue BLON.
Day 13	QUE 150 mg daily.	Marked improvement: erythema faded, papules significantly reduced.
Day 20 (follow-up)	QUE 150 mg daily.	Rash showed near-complete resolution.

QUE, quetiapine; BLON, blonanserin; EBA, ebastine; TAE, Triamcinolone Acetonide and Econazole Cream.

## 5 Outcome and follow-up

**Figure 3:**
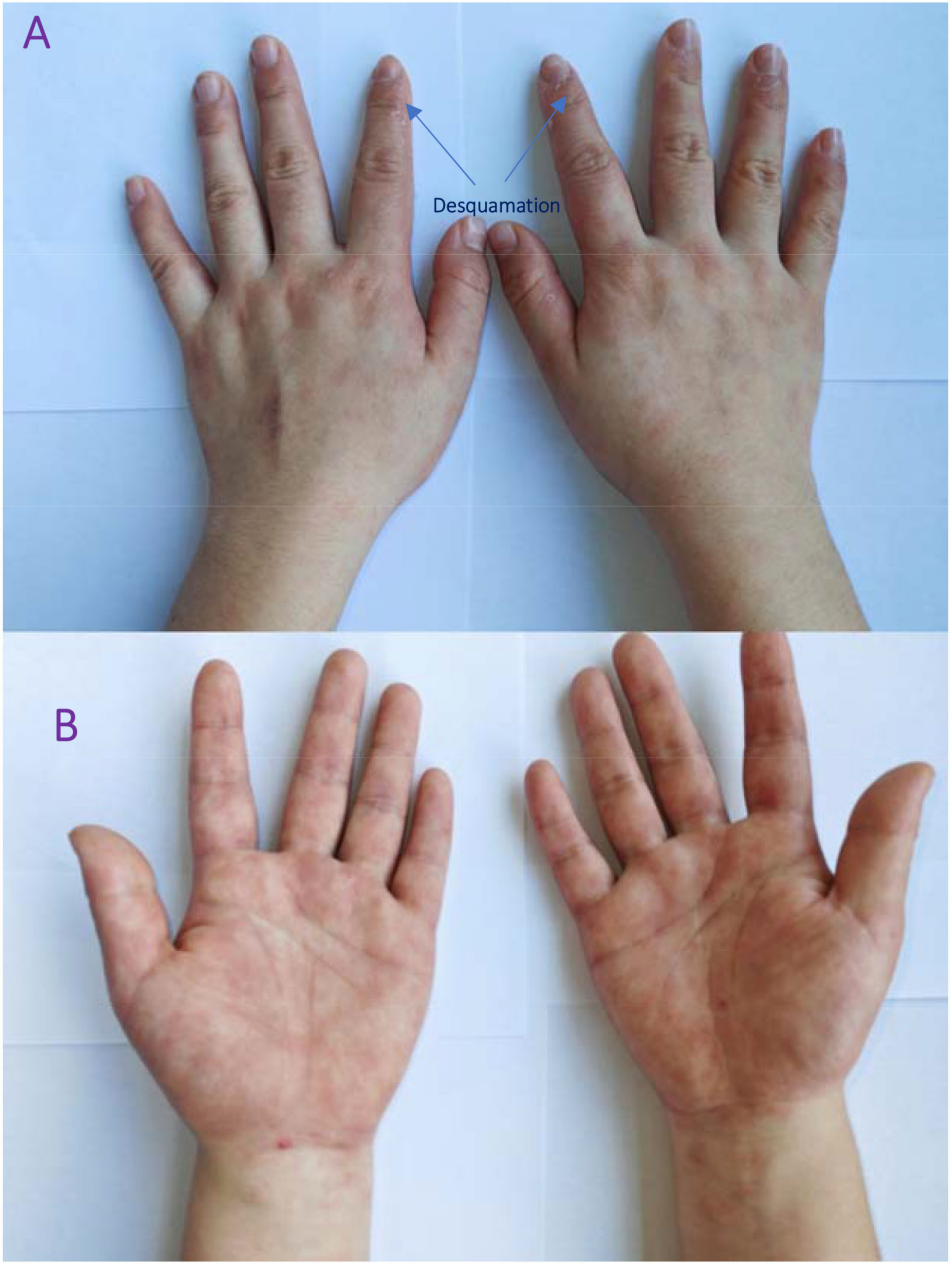
Improvement of the rash three days after blonanserin discontinuation. Fading erythema, significant reduction of papules, and early desquamation on the fingertips.

Within 72 hours of discontinuing blonanserin, the rash began to improve significantly, manifested by fading erythema and a reduction in papules. Significant improvement was observed 3 days after blonanserin discontinuation (**Figure 3**). The rash showed near-complete resolution one week after drug withdrawal.

## 6 Discussion

To our knowledge, this is the first report describing a bilateral hand-limited drug eruption in a patient receiving blonanserin. The adverse reaction was assessed using the Naranjo algorithm, which yielded a score of 5 (a score ≥ 5 denotes a ‘probable’ association) (8), confirming a probable causal relationship between the rash and blonanserin. Its localized distribution is distinct from the generalized exanthems previously reported with other second-generation antipsychotics (9). Importantly, the palmar involvement observed here is atypical, as the palms (and soles) are commonly spared in the most frequent type of drug-induced exanthem, the morbilliform or maculopapular rash. The mechanism underlying this unusual bilateral hand-limited pattern remains speculative. It may involve site-specific factors such as local drug or metabolite accumulation, though it is important to note that there is currently no evidence for the excretion of blonanserin or its metabolites in sweat, and eccrine gland density alone does not explain the dorsal hand involvement. Furthermore, confinement of the rash to the palms may also be related to early identification: The rash was detected on the 4th day of medication, still in the early stage of the immune response, before the drug or its metabolites reached the dose or distribution threshold sufficient to induce a generalized eruption; continued exposure might have led to extension to the limbs or trunk. Therefore, the localized presentation in this case can be seen as evidence that “early discontinuation” prevented progression.

To date, no published reports have linked the quetiapine-blonanserin combination to cutaneous adverse events of this nature. In our case, the temporal sequence and the positive ‘dechallenge’ outcome (with rash resolution upon blonanserin discontinuation despite quetiapine continuation) strongly implicate blonanserin as the sole causative agent. It is worth noting that the initial combination therapy was chosen to achieve rapid symptom control in an acute psychotic episode, leveraging blonanserin’s pharmacodynamic profile which is characterized by rapid D_2_ receptor occupancy (10), as an augmentative strategy to quetiapine monotherapy.

Mechanistically, blonanserin is metabolized by CYP3A4 into active intermediates, which can form haptens with skin proteins, subsequently activating a CD4^+^ T-cell-mediated immune response (11). The absence of systemic symptoms such as fever or eosinophilia argues against a DRESS-like reaction. The localized, bilateral hand involvement suggests a T-cell-mediated, site-specific inflammatory response, possibly triggered by localized drug accumulation or metabolite exposure.

Furthermore, the development of CADRs involves host-specific predisposing factors. Female sex is a recognized risk factor for various drug eruptions (12). Additionally, specific genetic polymorphisms, particularly in human leukocyte antigen (HLA) genes such as HLA-B*5801 and HLA-A*3101 (13), have been strongly associated with severe cutaneous adverse reactions to certain drugs. Although our patient did not undergo genetic testing due to ethical considerations, her profile—a young woman with a history of penicillin allergy—suggests a potentially heightened immune reactivity that may have contributed to her specific reaction pattern. This context enriches the understanding of individual susceptibility in blonanserin-related CADRs.

*In vitro* studies show that quetiapine is predominantly metabolized by cytochrome P450 (CYP) 3A4 (14). Similarly, blonanserin has also been identified as a substrate for CYP3A4 (15). Co-administration of two drugs sharing the same metabolic pathway increases the likelihood of competitive inhibition, which may elevate plasma concentrations and hasten the onset of adverse effects such as skin rash. Therefore, while the positive dechallenge strongly implicates blonanserin, a potential pharmacokinetic interaction with quetiapine contributing to the onset or presentation of the eruption cannot be entirely ruled out. Subsequent studies incorporating pharmacogenomic analysis could clarify whether HLA or other genetic markers modulate this risk.

This case re-emphasizes that prompt diagnosis, prompt identification, and early withdrawal of all suspect drugs are the critical first steps (16). Symptomatic treatment alone (antihistamines ± topical steroids) failed to control the condition, indicating that drug withdrawal remains the core measure when moderate-to-severe drug eruption is suspected. Psychiatrists should routinely inquire about skin changes within 4–6 weeks after initiating blonanserin and instruct patients to seek immediate medical attention if any new rash appears.

This case report has several limitations. First, the pathophysiological explanations for the hand-limited rash are hypothetical and lack confirmatory pharmacokinetic or histopathological data. Second, although a drug-drug interaction is considered unlikely given the clinical course, its contribution cannot be definitively excluded. Third, genetic testing for HLA or other susceptibility alleles was not performed. Finally, as a single observation, it cannot estimate the incidence of this reaction, and rechallenge with blonanserin was not ethically justifiable.

## 7 Conclusion

We report a case of bilateral hand-restricted rash most likely induced by blonanserin. Although rare, clinicians must be aware of this potential adverse reaction. A high index of suspicion, patient education, close monitoring after initiation, and immediate drug discontinuation upon rash appearance are crucial steps to ensure patient safety.

## Data Availability

The original contributions presented in the study are included in the article/Supplementary Material. Further inquiries can be directed to the corresponding author.
